# Supplementary material for: Multi-Level Model to Predict Antibody Response to Influenza Vaccine Using Gene Expression Interaction Network Feature Selection
Source: Microorganisms. 2019 Mar 14;7(3):79. doi: 10.3390/microorganisms7030079 (PMC6462975; doi:10.3390/microorganisms7030079)
Supplement: Supplementary file 1 [file microorganisms-07-00079-s001.pdf]

microorganisms

MDPI

Article

Multi-Level Model to Predict Antibody Response to Influenza Vaccine Using Gene Expression Interaction Network Feature Selection

Saeid Parvandeh [1], Greg A. Poland [2], Richard B. Kennedy [2] and Brett A. McKinney [1,3,*]

[1] Tandy School of Computer Science, University of Tulsa, Tulsa, OK 74104, USA; parvandehsaied@gmail.com
[2] Mayo Vaccine Group, Mayo Clinic, Rochester, MN 55905, USA; poland.gregory@mayo.edu (G.A.P.); kennedy.rick@mayo.edu (R.B.K.)
[3] Department of Mathematics, University of Tulsa, Tulsa, OK 74104, USA
* Correspondence: brett-mckinney@utulsa.edu; Tel.: +1-918-631-3444

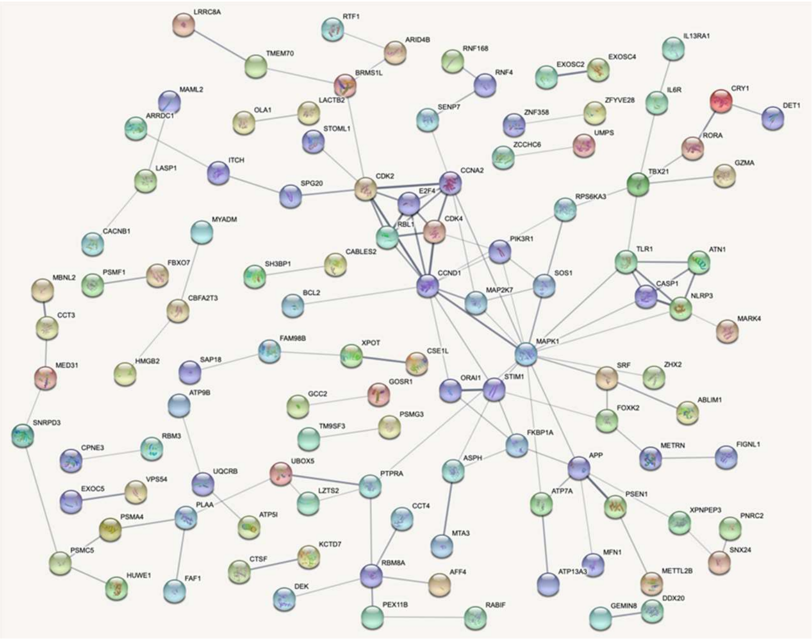

Figure S1. String network of top 200 genes used in analysis based on reGAIN matrix. Predicted interactions between genes based on text-mining of relevant publications. We hid unconnected genes. We found PPI enrichment *p*-value: 0.0012, number of edges: 92, average node degree: 0.92, and average local clustering coefficient: 0.326.

We compared the performance of the baseline models—baseline HAI alone (left column of Figure S2) and baseline HAI combined baseline gene expression (right column of Figure S2)—by plotting the observed versus predicted day 28 HAI fold changes. All models were trained on Baylor data (first row of Figure S2) and tested on Emory and Mayo data. When genes are included (right column of Figure S2), the predictions are more tightly correlated with the observed values, and the linear relationship between predicted and observed (blue line) becomes closer to the ideal relationship (red line).

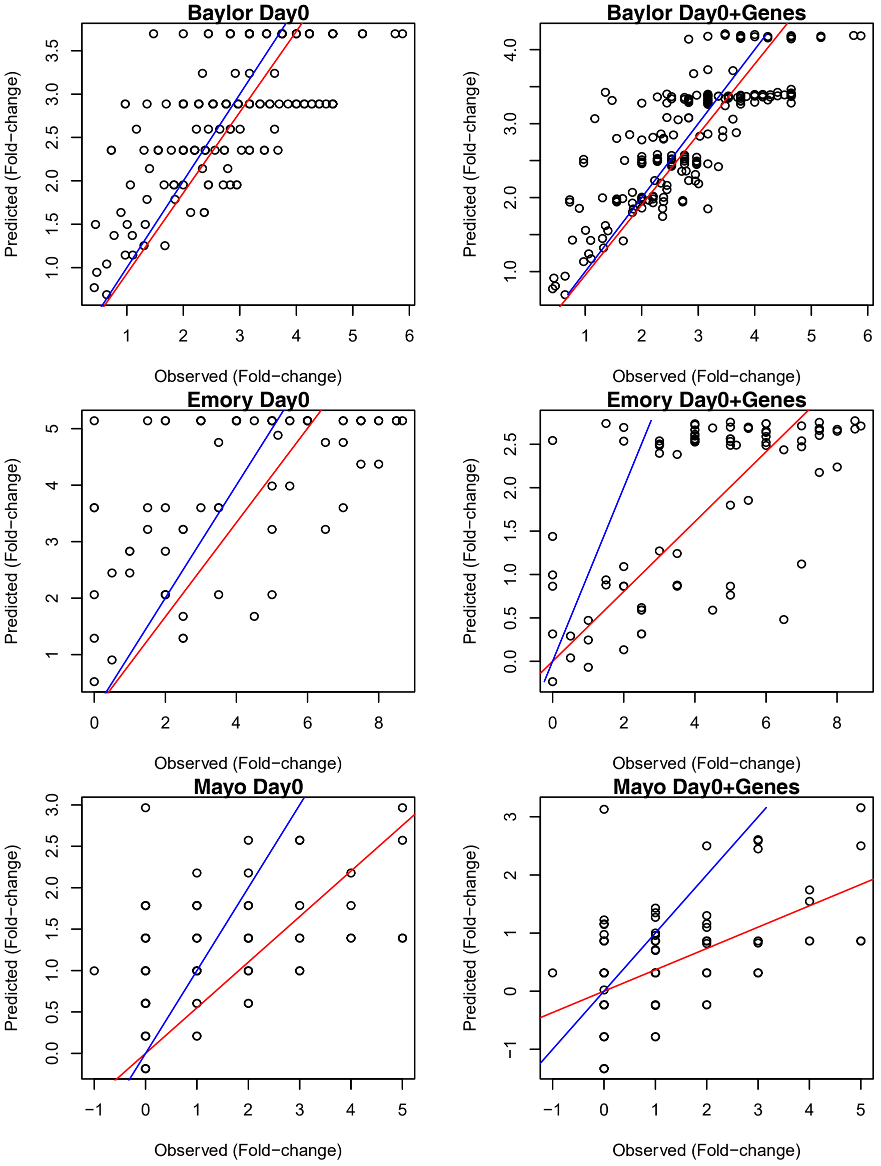

Figure S2. Panel of observed versus predicted (HAI plus gene model) plots of day-28 HAI fold changes for models that use only day-0 HAI as input (left plots) and models that include day-0 HAI and baseline gene expression (right plots). Subjects are combined from the three day-0 clusters (low day-0 HAI, medium day-0 HAI, and high day-0 HAI). The blue line is a 45° line depicting an ideal relationship between observed and predicted. The red line is a regression line without intercept between predicted and observed. Models were trained on the Baylor data (first row) and tested on Emory and Mayo.

microorganisms

MDPI

Table S1. Top 200 genes from Baylor data using nested cross-validation (CV) and regression-based Gene-Association Interaction Network (reGAIN) feature selection for all baseline HAI subjects.

Top 200 genes				
PEX11B	ZNF197	DLGAP4	UMPS	TLR1
HSF1	SPATA5	FKBP1A	RNF168	AHCTF1
ELMO2	STOML1	ATP7A	MAML2	LUZP1
PSMG3	MAPK1	THRAP3	SNRPD3	GCC2
ZNF517	PMM1	FOXK2	TSPAN13	RC3H1
CBFA2T3	TNRC6C	SERINC5	ZNF407	LASP1
SNX24	ARRDC1	IL6R	YY1AP1	DPF2
GRPEL1	RBM8A	QTRT1	DOK3	ST6GALNAC4
ZNF160	BBC3	PTPRA	VPS54	PANK3
THAP5	PJA1	EXOSC4	CPNE3	MPZL1
WDR37	COMMD6	ABLIM1	ASPH	CTDSPL2
MBNL2	SLC12A6	ZNF174	FBXO8	SOS1
LZTS2	CSE1L	MFN1	CCT4	RNF4
CHST11	TNIK	IGHMBP2	RORA	ATXN7L1
NLRP3	ATP5I	TXNL4B	CRTAM	PRKD2
ITCH	DDX20	ZNF430	FIGNL1	CHST14
ZCCHC6	DET1	PSEN1	CHCHD5	HMGB2
VEZT	PCGF5	ZHX2	ZC3H7A	ZNF44
MYO19	LRSAM1	LMBRD1	SOX12	PRPF38B
WDR25	APOBEC3D	OLA1	CDK2	TMEM70
GEMIN8	GZMA	MYADM	TM9SF3	PSMF1
ATN1	TBX21	RBL1	ZNF180	PIK3R1
FBXO7	ZNF142	XPOT	UBOX5	MNT
KCTD7	NOL10	TAF3	ORAI1	ATE1
MED31	ATP13A3	PWWP2B	LACTB2	SAP18
DNAJC10	CRY1	CCT3	METRN	ZNF589
ADAM19	GFM2	PPHLN1	RABIF	GPRIN3
SLC26A11	SRF	ATP9B	LONP1	GLI4
UQCRB	TAF9	RELL2	GOSR1	DEK
CDK4	EPHX1	HLCS	PARP3	HOOK3
MTA3	SH3BP1	RBM3	FAM98B	CASP1
RPLP1	FLNB	EXOSC2	PSMC5	SLC25A23
AFF4	ACBD6	RPS6KA3	RTF1	LRRC8A
TMEM41A	WDR44	CNIH4	ZFYVE28	HACE1
BRMS1L	XPNPEP3	ARID4B	RGS12	CTSF
CD36	BCL2	CABLES2	PNRC2	CENPL
FOXP4	MAPK13	IL13RA1	PSMA4	PLAA
ZNF358	MAP2K7	QPCTL	CCDC85B	RMND5B
SPG20	MARK4	CACNB1	METTL2B	CNOT10
EXOC5	HUWE1	SENP7	HDAC9	FAF1

© 2019 by the authors. Submitted for possible open access publication under the terms and conditions of the Creative Commons Attribution (CC BY) license (http://creativecommons.org/licenses/by/4.0/).
